# Hygienic quality of raw and fermented cow milk in the local milk sector of the Liptako-Gourma area in Niger

**DOI:** 10.14202/vetworld.2022.1541-1549

**Published:** 2022-06-25

**Authors:** Mariama Hima Gagara, Philippe Sessou, François S. P. Dossa, Paulin Azokpota, Issaka A. K. Youssao, Soumana Abdoulaye Gouro, Souaibou Farougou

**Affiliations:** 1 Communicable Diseases Research Unit (URMAT) of EPAC (Polytechnic School of Abomey-Calavi), University of Abomey Calavi, 01 BP 2009, Cotonou, Benin; 2 Central Livestock Laboratory (LABOCEL), BP 485 Niamey, Niger; 3 Food Formulations and Molecular Biology Laboratory, Faculty of Agronomic Sciences, University of Abomey-Calavi, 01 BP 526 Cotonou, Benin; 4 Faculty of Agronomy (FA), Abdou Moumouni University of Niamey, BP 10960 Niamey, Niger

**Keywords:** bovine, fermented milk, microbiological quality, milk, physical parameters

## Abstract

**Background and Aim:**

Milk is a food of high nutritional value, which occupies an undeniable place in the human food ration, but is an ideal medium for microbial growth. This study aims to assess the hygienic quality of local raw and fermented milk from the Liptako-Gourma region in Niger.

**Materials and Methods:**

We performed physical and bacteriological analyses on 330 samples of bovine milk from local breeds, including 110 individual milk samples (per cow), 110 fermented milk samples, and 110 blended milk samples. The microbiological parameters were determined using standard methods.

**Results:**

The physical analysis revealed temperatures during sample collection for all milk types between 35.2°C and 37.8°C. The average pH of fermented milk varied between 3.16 and 4.92 and those of individual and blended raw milks between 5.42 and 6.98. The titratable acidity varied from 15° to 18.1°D for raw milk and between 59° and 122°D for fermented milk. The average density of individual and blended milks ranged between 1.028 and 1.035. Regionally, milk samples from Tillaberi had a significantly higher aerobic mesophilic germ (GAM) load (7.42 ± 0.53 × 10^7^ Colony-forming unit/mL; p = 0.0025) compared to the Dosso and Niamey regions. The prevalence of *Staphylococcus aureu*s, *Escherichia coli*, and *Salmonella* spp. were 86.36%, 12.73%, and 20.91%, respectively, in fermented milk. Phenotypic identification pointed toward three genera: *E. coli* (30.76% ± 0.25%), *S. aureus (*20.58% ± 0.14%), and *Salmonella* spp. (2.74 ± 0.04%).

**Conclusion:**

The present data suggest that milk samples collected from three regions in Liptako-Gourma had low quality; further, some of the bacteria identified (*E. coli*, *S. aureus*, and *Salmonella* spp.) could be potential foodborne pathogens.

## Introduction

Milk and dairy products have long been a key element in the human diet because they provide a matrix of nutritional compounds, including fats, proteins, vitamins, antioxidants, and minerals [1, 2]. In addition to their nutritional value, the consumption of dairy products is also associated with health benefits [3]. Simultaneously, a recent report by the Center for Disease Control stated that consumption of raw dairy products is linked to foodborne illness; dairy products are the second main source of pathogenic microorganisms for humans, causing 14% of foodborne illnesses and 10% of deaths [4]. Indeed, milk is a fragile product, liable to be altered by numerous chemical, biochemical, and microbiological reactions if not well preserved [5]. Raw or processed milk is an excellent culture medium for several microorganisms [6] and can result in product spoilage or infections/poisoning among consumers [7].

In West Africa, sustained population growth and the emergence of a middle class have significantly increased the demand for dairy products [8]. In Niger, animal products, including milk, contribute to meeting the nutritional requirements of the population [9]. However, direct milk sale to the consumer, which is the most frequent form of acquisition, presents a risk due to the defective quality of milk and dairy products [10]. In fact, other developing countries are also paying a heavy price for unsafe food products. The World Bank estimates that food safety problems cost developing countries >$100 billion a year. Despite existing standards to preserve milk quality in Niger, including Law No. 2004-048 of June 30, 2004, on the framework law related to breeding [11] or the Niger standard on milk and fermented milk products of December 2006 [12], Decree No. 2011-616/PRN/MEL of November 25, 2011, regulating the hygiene inspection of animal and animal-related food products [13], there is a still an issue with milk quality. Milk contamination is closely dependent on hygienic conditions [14]. Milk quality, considered with respect to bacterial contamination, largely depends on the observance of sanitary standards at all stages (milking, processing, storage, and transportation) [15]. The previous studies [9, 15] in some regions in Niger reported poor hygiene in dairy farms. The lack of hygiene in milk production increases the risk of microorganism proliferation. Moreover, since the quality of raw milk influences that of dairy products [16], it would be important to know the quality of the local milk used to generate the country’s dairy products. This knowledge would also improve the market value of local milk production. However, data on the microbiological quality of milk are scarce in Niger [17, 18]. The most recent data relates to germs linked to mastitis and bovine tuberculosis [19, 20].

This study aimed to investigate the hygienic quality of local milk, which may support the country on its journey toward its Zero Hunger objective by 2030.

## Materials and Methods

### Ethical approval

Ethical approval was not required for this study; however, milk samples were collected as per the standard collection procedure without any harm to the animals.

### Study period and location

The study was conducted from March to September 2015 in the Liptako-Gourma area in Niger. This area is made up of 3 regions of the country, including Dosso, Niamey, and Tillabery, in which 11 municipalities were selected, each corresponding to a dairy basin. In the area of Liptako-Gourma, breeding represents the second economic activity of the country. The Azawak breed is the most exploited [21], and produces an average of 10 L of milk/day in 6 months of lactation [22]. However, its average production does not exceed 3 L/day [23]. Indeed, the choice of this area is justified by its vast water network, which is a fundamental factor for animal production, the genetic potential of the Azawak (one of the most exploited breeds in this area but also one of the best dairy of the sub-region) and the presence of the country’s capital (Niamey) where the demand for dairy products is the highest. For the study, 11 dairy basins were selected namely, Hamdallaye, Baleyara, Karma, Torodi, Tillaberi, Say, Sansane-Haoussa, Kollo, Niamey, Dosso, and Birni N’Gaouré.

### Sample collection

A cross-sectional study was carried out to assess the microbiological quality of raw and fermented milk samples from the three major players (producers, collectors, and sellers) in the local milk sector in the Liptako-Gourma area. Samples were collected aseptically at a rate of 500 mL of raw milk directly from the cow’s udder (individual), 500 mL of mixed milk (from collection points), and 500 mL of fermented milk (from markets). The samples were packaged in sterile Pirex bottles^®^ (Thermo Fisher Scientific, France) and transported in coolers fitted with frozen carbo glass to the laboratory. The samples were analyzed within 24 h.

### Sampling sites

Sampling concerned individual milk at the farm level (producers), blending milk at the farm and collector level, and fermented milk at the vendor level (Table-1). Each site was visited only once. These products are made from local milk and are the most consumed in target localities. In each of the three regions, the farms were selected by iterative sampling guided by resource persons (snowball sampling), where the first was selected at random from a list provided by the local authorities [24]. The farm selection was based on the following criteria:

**Table 1:** Distribution of the number of samples taken by dairy basins.

Regions	Dairy basins	Individual milk	Blending milk	Fermented milk	Total
Niamey	Niamey	10	10	10	30
Dosso	Dosso	10	10	10	30
	BirniN’Gaoure	10	10	10	30
Tillaberi	Hamdallaye	10	10	10	30
	Baleyara	10	10	10	30
	Karma	10	10	10	30
	Torodi	10	10	10	30
	Say	10	10	10	30
	Sansane-Haoussa	10	10	10	30
	Tillabéri	10	10	10	30
	Kollo	10	10	10	30
Total		110	110	110	330

- Farms primarily used for milk production
- Farm with ≥10 lactating cows
- Cooperation and acceptance of the breeder to participate in the study and allow the collection of milk samples

To select collectors, the collection points in each dairy basin were identified with the help of local authorities. The first collectors to arrive and who have given their consent for the collection of milk samples were selected. Fermented milk was acquired from the points of sale in the localities of the selected farms. A total of 330 samples were taken from the 11 regions selected at a rate of 30 samples per region.

### Physical analysis

The temperature and pH of raw and fermented milk samples were measured using a pH meter branded EXTECH/Exstik™ pH Meter thermometer (PH105-PH Module; CL205-Chlorine Module; RE305-ORP Module). A sufficient milk quantity to cover the immersed electrode was transferred into beakers for temperature and pH measurements. The density was determined using a thermolacto-densimeter (PAAR DMA 35) at 20°C. The acidity was measured by titration with NaOH solution and expressed in Dormic degrees (°D).

### Microbiological analysis

The isolation of various microorganisms in all raw and fermented milk samples was carried out according to International Organization for Standardization (ISO) 7218 standards in food microbiology [25]. In this study, we investigated the total aerobic flora at 30°C, thermotolerant coliforms and *Escherichia coli* at 44°C, *Staphylococcus aureus*, sulfite-reducing anaerobic germs at 37°C, *Clostridium perfringens* at 46°C, *Salmonella* spp., *Listeria monocytogenes*, yeasts, and molds. Suspected pathogenic strains were isolated and biochemically identified using analytical profile index galleries which are a biochemical panel for identification and differentiation of members of the family of bacteria and yeasts (Table-2).

**Table 2:** Culture media used for the isolation of microorganisms in milk.

Microorganisms	Identification media/References	Incubation (Temperature °C/Time Hours)		Standards	Methods
Mesophilic aerobic flora	PCA/Biokar BK144HA - Solabia Group France	30°C	72 h	NF EN ISO 4833-1: 2013	Enumeration on PCA
Total coliforms	Crystal VRBL/Biokar BK152HA - Solabia Group France	30°C	24 h	NF ISO 4832, 2006	Enumeration on VRBL, Biochemical confirmation by API 20 E gallery
*Escherichia coli*	Rapid’ *E. coli*/Bio-Rad 355 5299 - Bio-Rad Laboratories	37°C	24 h	EN ISO 16140, 2003	Isolation of strains on Rapid’ *E. coli*, Biochemical confirmation by API 20 E gallery
Sulfite-reducing anaerobes	TSC/Biokar BK031HA - Solabia Group France	37°C	24 h	NF EN 13401, 2003	Enumeration on TSC medium, Biochemical confirmation by API 20A gallery
*Staphylococcus aureus*	Baird Parker/Biokar BK055HA - Solabia Group France	37°C	24 h	NF EN ISO 6888-1, 1999	Enumeration on Baird Parker agar medium, Confirmation by coagulase test, Biochemical confirmation by Galerie API Staph
*Listeria monocytogenes*	*“Fraser-half” broth/OxoidCM 1053B - Oxoid Group Thermo Fisher Europe *Fraser broth/Oxoid CM 0895 - Oxoid Group Thermo Fisher Europe *Palcam/Oxoid CM 0877 - Oxoid Group Thermo Fisher Europe *TSYEA/Conda 1398 - Conda Laboratories Europe	30°C 37°C 37°C 37°C	24 h 24 h 48 h 24 h	NF EN ISO 11290-2 (1998)	Selective enrichment with “Fraser -demi,” Enrichment with Fraser Broth Isolation on Palcam Isolation on TSYEA Biochemical confirmation by API Listeria gallery
*Salmonella* spp*.*	Buffered peptone water (Eur Pharm) Conda - Conda Laboratories Europe	37°C	24 h	NF EN ISO 6887-5: 2010	Pre-enrichment in buffered peptone water
	Rappaport-Vassiliadis/Conda 1240.00 - Conda Laboratories Europe	37°C	24 h	ISO 6579 2002	Selective enrichment with Rappaport-Vassiliadis
	SS (Conda 1064.00)/Xylose-Lysine-Deoxycholate (Conda 610060) - Conda Laboratories Europe	37°C	24 h		Isolation on XLD and SS medium. Biochemical confirmation by API 20 E.
Yeasts and molds	Sabouraud with chloramphenicol/Conda 1090.00 - Conda Laboratories Europe	30°C	72 h	ISO 6611: 2004 (IDF 94: 2004)	Enumeration on Sabouraud Chloramphenicol Agar
Lactic acid bacteria	MRS agar/Oxoid CM 361-Oxoid Group Thermo Fisher Europe MRS broth/Oxoid Group Thermo Fisher Europe	37°C	72 h	NF ISO 15214, 1998	Enumeration on MRS agar Isolation on MRS Broth Biochemical confirmation by API 50CH
	M17 agar/Conda 1318.00 - Conda Laboratories Europe M17 broth/Oxoid CM 0817 - Oxoid Group Thermo Fisher Europe	37°C 37°C	24 h 24 h	ISO 7889/IDF, 2003	Enumeration on M17 agar Isolation on M17 Broth Biochemical confirmation by API 50CH

PCA=Plate Count Agar, VRBL=Violet and Neutral Red Bile Lactose Agar, TSC=Tryptone Sulfite Cycloserine, TAYEA=Tryptose Soy and Yeast Extract, MRS=Man Rogosa Sharpe, XLD=Xylose-Lysine-Deoxycholate, SS=Salmonella-Shigella, ISO=International Organization for Standardization

### Preparation of the stock suspension and dilutions

Sample dilutions were performed according to ISO 8261 [26]. Of the 500 mL of milk, 25 mL from each unit were diluted in an Erlenmeyer flask containing 225 mL of buffered peptone water pH 7.0 ± 0.2 (Eur Pharm) Conda) to obtain a stock dilution at 10^−1^. Decimal dilutions were made by placing 1 mL of each dilution in a tube containing 9 mL of tryptone salt up to 10^−7^.

### Statistical analysis

The data collected were entered into Microsoft Excel (Microsoft, USA), and then analyzed with Statistical Analysis System (SAS) 9.4. software (SAS Institute Inc., NC, USA). For physical parameters (temperature, density, acidity, and pH), a single-factor analysis of variance was used with collection region and type of milk collected as a source of variation. The Proc General Linear Model (GLM) was used for the analysis of variance and the F test was used to determine the importance of region and type of milk on the variables. The means were calculated and compared in pairs by Student’s t-test. For microbiological parameters (Mesophilic aerobic flora, Lactic bacteria, *E. coli, S. aureus*, Yeasts/Molds, Sulfite reducing anaerobes, and *L. monocytogenes*), microbial counts were converted to log base–10 of the number of Colony-forming units (CFUs) per mL of raw cow’s milk (log CFU/mL). Means and standard deviations were calculated. Data were analyzed with ANOVA using the GLM procedure of the SAS software; the least significant differences were used to separate the averages at p < 0.05. P indicates the relative frequency and N the sample size. Frequencies were calculated by the SAS Proc freq procedure and compared by the Chi-square test (a = 0.05) and bilateral Z test (a = 0.05).

## Results

### Physical analysis

The average temperature ranged between 35.2°C and 37.8°C for all types of milk. The average pH of fermented milks ranged between 3.16 and 4.92; 57.27% (62.99/110) complied with the pH standard (4–4.5). The pH of individual and blended milks ranged between 5.81 and 6.96 and between 5.42 and 6.98, respectively. However, only 21.81% (24.06/110) of these individual milks and 16.36% (17.99/110) of blended milks were within the standard pH range of normal raw milk (6.6 < pH < 6.8). The titratable acidity ranged between 15–17.9°D, 15–18.1°D, and 59°–122°D for individual milk, blended milk, and fermented milk, respectively. However, there were no significant differences (p > 0.05) between milk types. The average density of individual and blended milks ranged between 1.028 and 1.033 and between 1.030 and 1.035, and showed significant differences (p ≤ 0.0001). Regarding density, 96.36% of individual milks and 91.81% of mixed milks were not wet, being within the normal density standard for raw milk (1.030–1.034). All physical parameters are reported in Table-3.

**Table 3:** Average values of temperature, pH, density, and titratable acidity of single milk, blended milk, and fermented milk according to region.

Variables	Temperatures in °C (M ± ES)			

	Dosso	Niamey	Tillabéri	Significance
Fermented milk	36.89 ± 0.45^a^	37.15 ± 0.52^a^	36.86 ± 0.39^a^	NS
Individual milk	36.86 ± 0.46^a^	37.10 ± 0.48^a^	36.88 ± 0.53^a^	NS
Blending milk	37.00 ± 0.41^a^	36.95 ± 0.42^a^	36.85 ± 0.58^a^	NS

**Variables**	**pH (M ± ES)**			

	**Dosso**	**Niamey**	**Tillabéri**	**Significance**

Fermented milk	4.15 ± 0.35^a^	4.25 ± 0.24^a^	4.23 ± 0.44^a^	NS
Individual milk	6.54 ± 0.13^a^	6.52 ± 0.33^a^	6.51 ± 0.20^a^	NS
Blending milk	6.49 ± 0.25^a^	6.47 ± 0.29^a^	6.44 ± 0.40^a^	NS

**Variables**	**Density (M ± ES)**			

	**Dosso**	**Niamey**	**Tillabéri**	**Significance**

Individual milk	1.03 ± 0.00	1.03 ± 0.00	1.03 ± 0.00	*
Blending milk	1.03 ± 0.00^a^	1.03 ± 0.00^a^	1.03 ± 0.00^a^	NS

**Variables**	**Acidity in °D (M ± ES)**			

	**Dosso**	**Niamey**	**Tillabéri**	**Significance**

Fermented milk	80.02 ± 18.90^a^	73.6 ± 12.41^a^	77.86 ± 17.34^a^	NS
Individual milk	16.43 ± 0.60^a^	16.63 ± 0.64^a^	16.44 ± 0.65^a^	NS
Blending milk	16.23 ± 0.75^a^	16.10 ± 0.90^a^	18.39 ± 15.70^a^	NS

NS=Not significant, M ± ES=Average plus or minus Standard Error,

* Significant at the 0.05% level. a=no significant difference for means followed by the same letter. The means of the same column followed by different letters are significantly different at the 5% level

### Microbiological analyses

#### Microbial loads of milk within regions

All parameters investigated in the milk samples from the three regions varied (Table-4). For germs indicative of overall hygiene (Mesophilic aerobic germs), milk in the Tillaberi region was significantly more contaminated (p = 0.0025). Lactic acid bacteria, coliforms, and Staphylococci were also found in greater proportion in milk from Tillaberi, although the differences between regions were not significant. Milk samples from the Niamey region were the most contaminated with yeasts and molds. *Salmonella* was absent from milk samples taken in the Niamey region. However, it was present in 20% (22/110) of milk samples from Dosso and in 15% (16.5/110) from Tillaberi.

**Table 4:** Average values of the microorganisms counted in the milk according to the regions.

Variables	Average values (M ± ES)			

	Dosso	Niamey	Tillabéri	Significance
GAM (10^7^ CFU/mL)	7.28 ± 0.38^b^	7.24 ± 0.16^b^	7.42 ± 0.53^a^	**
Lactic acid bacteria (10^5^ CFU/mL)	6.48 ± 0.33^a^	6.53 ± 0.23^a^	6.54 ± 0.33^a^	NS
Coliforms (10^3^ CFU/mL)	5.52 ± 0.85^a^	5.76 ± 0.70^a^	6.13 ± 0.50^a^	NS
*Staphylococcus aureus* (10^4^ CFU/mL)	3.54 ± 1.89^a^	3.58 ± 1.83^a^	3.85 ± 1.63^a^	NS
Yeasts (10^5^ UFC/mL)	6.53 ± 0.27^a^	6.57 ± 0.25^a^	6.50 ± 0.38^a^	NS
Molds (10^5^ UFC/mL)	6.48 ± 0.30^a^	6.55 ± 0.25^a^	6.40 ± 0.40^a^	NS

GAM: Aerobic Mesophilic Germs; NS=Not significant, M ± ES=Average plus or minus Standard Error,

** Significant at the 0.01% level. a and b=Significant difference for means followed by the same letter. The means of the same row followed by different letters are significantly different at the 5% level

#### Microbial loads

The average total flora ranged between 7.36 ± 0.49 and 7.39 ± 0.51 × 10^7^ CFU/mL. Fermented milks exhibited the highest concentrations, followed by blended milks and individual milks (Table-5). They also had the highest average load of lactic acid bacteria (average between 6.51 ± 0.33 and 6.55 ± 0.29 × 10^5^ CFU/mL), yeast (average between 6.51 ± 0.33 and 6.52 ± 0.37 × 10^5^ CFU/mL), and molds average (6.40 ± 0.34 and 6.44 ± 0.40 × 10^5^ CFU/mL). Blended milks were the most contaminated, with coliforms with an average load between 5.94 ± 0.70 and 6.02 ± 0.62 × 10^3^ CFU/mL and average *S. aureus* loads from 3.54 ± 1.89 to 3.92 ± 1.57 × 10^4^ CFU/mL. However, no significant difference was observed between milk types regarding microbial loads (Table-5).

**Table 5:** Average values of the microorganisms counted according to the various types of milk.

Variables	Average values (M ± ES)			

	Fermented milk	Individual milk	Blending milk	Significance
GAM (10^7^ CFU/mL)	7.39 ± 0.51^a^	7.36 ± 0.49^a^	7.38 ± 0.47^a^	NS
Lactic acid bacteria (10^5^ CFU/mL)	6.55 ± 0.29^a^	6.51 ± 0.33^a^	6.52 ± 0.33^a^	NS
Coliforms (10^3^ CFU/mL)	6.00 ± 0.62^a^	5.94 ± 0.70^a^	6.02 ± 0.62^a^	NS
*Staphylococcus aureus* (10^4^ CFU/mL)	3.70 ± 1.76^a^	3.70 ± 1.76^a^	3.92 ± 1.57^a^	NS
Yeasts (10^5^ UFC/mL)	6.52 ± 0.37^a^	6.51 ± 0.36^a^	6.51 ± 0.33^a^	NS
Molds (10^5^ CFU/mL)	6.44 ± 0.40^a^	6.40 ± 0.38^a^	6.40 ± 0.34^a^	NS

GAM=Aerobic Mesophilic Germs, NS=Not significant, M ± ES=Average plus or minus Standard Error, a=no significant difference for means followed by the same letter

With respect to the prevalence of the pathogenic microorganisms (Table-6), fermented milks were the most contaminated, with 86.36% ± 0.06% *S. aureus*, 12.73% ± 0.06% *E. coli*, and 20.91% ± 0.08% *Salmonella* spp. Sulfite-reducing anaerobes equally predominated in fermented and individual milks, while *L. monocytogenes* was found most commonly in blending and individual milks.

**Table 6:** Level of contamination of milk by pathogenic microorganisms.

Microorganisms	Level of pathogen presence in the types of milk (% ± IC)			Significance

	Individual milk	Blending milk	Fermented milk	
*Staphylococcus aureus*	81.82 ± 0.07^a^	81.82 ± 0.07^a^	86.36 ± 0.06^a^	NS
*Listeria monocytogenes*	4.55 ± 0.04^a^	13.64 ± 0.06^a^	13.64 ± 0.06^a^	NS
Sulfite-reducing anaerobes	10 ± 0.06^a^	9.09 ± 0.05^a^	10 ± 0.06^a^	NS
*Escherichia coli*	3.64 ± 0.03^a^	7.27 ± 0.05^a^	12.73 ± 0.06^a^	NS
*Salmonella* spp*.*	10.91 ± 0.06^a^	13.64 ± 0.06^a^	20.91 ± 0.08^a^	NS

IC = Correlation index, NS = Not significant, a = no significant difference for means followed by the same letter

Biochemical identification of suspected pathogenic strains (Table-7) confirmed the presence of 30.76% (4/13) *E. coli*, 20.58% (7/34) *S. aureus*, and 2.74% (2/73) *Salmonella* spp.

**Table 7:** Percentage of positive for pathogenic microorganisms.

Pathogenic microorganisms	Number of isolates	Suspected	Percentage of positives	IC
*Staphylococcus aureus*	215	34	20.58% (7)	0.14
*Salmonella* spp.	73	73	2.73% (2)	0.04
*Escherichia coli*	26	13	30.76% (4)	0.25

IC = Correlation index

## Discussion

In the present study, we found that the temperature of raw milk (individual and mixed) and even that of fermented milk was close to body temperature (37°C), which is the average temperature during the study period (March to September). This shows that after milking, milk does not immediately arrive at the collection center having time to acclimate. Fermented milk (the most widely consumed dairy product) mostly drinks alone or mixed with processed cereal (porridge, scoop, and dengue) as a midday or evening meal. The times of high demand coincide with the maximum temperature, which accentuates milk acidification, explaining the low pH observed. The lack of freshness of certain milk samples might be attributable to a long time after milking and storage at an inappropriate temperature, conditions conducive to the transformation of lactose into lactic acid by lactic acid bacteria [27, 28]. However, the amount of acid produced by bacteria is a determining factor in milk quality, as is tampering. Indeed, pH and acidity depend on hygienic conditions during milking, the initial total microbial flora and its metabolic activity and milk handling [29, 30]. The fact that we found standard densities is proof that dairy producers do not adulterate milk intended for sale.

Microbiological analysis revealed that microbial loads depend on the nature of the product analyzed and the microorganism investigated.

The mesophilic aerobic germ load was very high in all milk samples from the three regions being over the 10^6^ CFU/mL acceptable threshold. The total aerobic mesophilic flora provides information on milk’s hygienic quality [31], due to its relationship with non-compliance with good milk production and storage practices [32]. Non-compliance with the standard may render the product unfit for human consumption even in the absence of pathogenic flora [33]. Our result confirms the faulty hygiene observed in the practices of dairy farmers in the Liptako-Gourma region in Niger [9]. However, it should be noted that hygienic practices differ from region to region. The greatest overall microbial loads recorded in the Tillaberi region would result from the long time elapsed before the milk reaches the consumer. Due to the vastness of the region, production areas are not only far from each other but also from consumption areas, which increases the delivery time. The lowest contamination levels in Niamey would comply with hygienic rules. The hot weather does not help with milk conservation [34]. In fact, in Niger, high temperatures at certain times of the year contribute to the rapid deterioration of food in general and dairy products in particular and can increase the presence of pathogenic germs [34]. While temperature control is a big issue, adhering to hygienic production practices could drastically lower the microbial load in milk. Considered a flora of technological interest [35], but also of alteration [36], fungal flora can degrade product marketability by altering taste, odor, and appearance. The results of our study show that this flora is present in all types of milk but more so in fermented milk, probably due to fungi’s acidophilic nature and their low sensitivity to antagonistic lactic acid bacteria [37]. The massive presence of fungal flora is also the expression of strong external contamination and poor tool hygiene [38]. The presence of coliforms in all dairy products reflects poor hygienic conditions during production and an unhealthy environment because coliforms are usual hosts in mammal intestines and their presence in milk indicates fecal contamination [39**]**. Coliforms of the genus *Escherichia* contaminate milk directly (through the udder), or multiply as a result of improper cleaning of utensils [40], which could explain the higher microbial load in raw blended milks. The highest coliform load in blended milk would be due to the numerous manipulations by milk collectors together with the various unhygienic manipulations of dairy producers (washing of hands, teats, and utensils with water of questionable quality), which, instead of guaranteeing hygiene contaminate the hands, udder, and equipment. However, if fermentation can cause sufficient milk acidification (pH < 4.5) would impair their growth since these bacteria are not acidophilic. An exception is *E. coli* O157: H7, which can resist the acidic environment [41] and survive for up to about 4 weeks [42]. Another possibility might be insufficient acidification during fermentation [43]. Most *E. coli* strains are harmless, coexist with the host in the intestinal tract, and may benefit the host by protecting it against infection by pathogenic bacteria [44] and by synthesizing vitamin K in the host’s intestine. However, some can cause disease by influencing the host or acquiring virulence. Among these pathogenic strains, enterohemorrhagic *E. coli* (EHEC) producing Shiga toxins are particularly dangerous, causing disastrous health and economic consequences as the Shiga-toxin kills host cells in the intestine and can enter the bloodstream to affect other organs, such as the kidneys and brain causing increased complications compared to other bacterial causes of gastroenteritis [45]. *S. aureus* contaminates milk either by direct excretion from the udders of animals with mastitis or during the handling and processing of raw milk [46]. The numerous manipulations (milking and decanting) by multiple actors could increase the presence of this pathogen in blended milk samples. *S. aureus* is a cause of collective food poisoning [47], and worse still, it can produce heat-stable enterotoxins [48]. Fortunately, this strain is not resistant to disinfectants, except in the case of biofilm formation [49]. *Salmonella* was found in all types of milk, further attesting the poor hygiene during production and processing. On a farm, the main sources of *Salmonella* are the feces of sick animals or asymptomatic carriers. Salmonella is spread through water, solid feed, or excreta from one farm to another. When the material lays on the ground during milking, the milk can get contaminated by *Salmonella* spp., especially since even the water used for washing could also be contaminated [50]. As for *Salmonella*, they are generally transmitted to humans by ingestion of contaminated food and are responsible for diseases such as typhoid fever, paratyphoid fever, and salmonellosis, among other non-typhoid ones, which most often lead to gastroenteritis [51].

Analyzing the water used in the farms and for dairy processing would have made allowed verifying the role of water quality in contamination. It would also be interesting to establish the link between risky practices and product quality while identifying the critical points to ensure the sanitary quality of these products. In addition, milk quality would be improved by respecting good production practices, good hygiene practices, respecting the heat treatment process, washing utensils with soap or bleach and good quality water, reducing transport time, use of lactoperoxidase, and mastery of fermentation processes. The finding of certain pathogenic species in milk represents a real threat to public health. Given the lacking process, environmental, and material hygiene, it is imperative to investigate the incidence and effects of the presence of these pathogenic microorganisms in milk.

## Conclusion

The results of physical and microbiological analyses of raw and fermented cow’s milk samples demonstrated the presence of microorganisms such as *E. coli*, *Salmonella* spp., and Staphylococci, showing unsatisfactory hygienic quality. High and variable bacterial loads result from variability in production, collection, and processing practices. These products imply risks to consumers’ health. This is the first study in the region unveiling the real risk incurred by consumers and also investigates the acidic level that inhibits pathogen growth. To guarantee food safety and improve the hygienic quality of milk and fermented milk, good hygiene practices and periodic microbiological control are an absolute necessity. Indeed, the absence of adequate regulations and product control to ensure sufficient hygienic quality of the dairy products marketed results in unsafe products. It is essential to raise awareness and train, individually or collectively, all players in the sector in hygienic rules and transformation processes. The concerted intervention of involved players in the sector considering their respective needs combined with incentive measures would also be desirable.

## Authors’ Contributions

MHG, PS, and SF: Conceptualization and experimental design. MHG and FSPD: Collected milk samples, provided laboratory technical support, conducted the experiments, analyzed data, and drafted the manuscript. PS, PA, IAKY, SAG, and SF: Supervised the study and reviewed and edited the manuscript. All authors have read and approved the final manuscript.
